# Lepidic and alveolar subepithelial squamous cell carcinoma: expansion of the concept of peripheral squamous cell carcinoma with proposal for revised terminology based on morphologic, immunophenotypic, and clinical analysis of 22 cases

**DOI:** 10.1007/s00428-026-04524-z

**Published:** 2026-04-23

**Authors:** Federica Filipello, Francesca Ambrosi, Hans Blaauwgeers, Johanna Grefte, Wim Vos, Luisella Righi, Erik Thunnissen, Teodora Radonic

**Affiliations:** 1 Department of Pathology, San Luigi Hospital, Orbassano, TO Italy; 2 DIAP-Dipartimento Interaziendale Di Anatomia Patologica Di Bologna, Pathology Unit, Maggiore Hospital- AUSL Bologna, and Department of Medical and Surgical Sciences (DIMEC), University of Bologna, Bologna, Italy; 3 Department of Pathology, OLVG LAB BV, Amsterdam, The Netherlands; 4 Department of Pathology, Gelre Hospitals Apeldoorn and Zutphen, Apeldoorn, The Netherlands; 5 Department of Pathology, Amsterdam University Medical Center, Amsterdam Cancer Center, Amsterdam, The Netherlands; 6 Department of Oncology, University of Turin at San Luigi Hospital, Orbassano, TO Italy

**Keywords:** Squamous cell carcinoma, Subepithelial, Lung carcinoma, Subsolid tumour

## Abstract

In recent years, peripheral type SCC with alveolar filling growth pattern was described with an increasing prevalence in up to 55% of patients. We describe a series of rare growth pattern of SCC with subtotal or partial growth of tumor cells under reactive type II pneumocytes, termed subepithelial growth. Retrospective case series of 19 resections specimens and 3 biopsies with subepithelial growth of SCC and clinical and radiological correlation is described. All 19 resected SCC showed in histology beside a central invasive component subtotal or partial growth of tumor cells under reactive type II pneumocytes (subepithelial component 10–100%). Immunohistochemistry (IHC) for TTF1/p40 double stain revealed p40 + /TTF1- in the tumor cells and p40-/TTF1 + in the pre-existing epithelial cells. IHC for p53 demonstrated in 91% cases mutation type labeling in the p40 + tumor cells and in 100% cases a wild-type pattern in TTF1 + cells. On CT-scan four cases with subtotal subepithelial component ≥50% presented as part solid nodule with a peripheral ground glass component. Most of tumors were peripheral (91%) and pTNM ranged from pT1bN0M0 to pT3N2M0. In 71% patients with available clinical information, progression free survival (PFS) was 100% after a median follow up of 20 months (range 4–56 months). In 19% of patients association with interstitial lung disease was observed. SCC may spread in a subepithelial manner along alveolar septa under pneumocytes type 2 and could have a corresponding peripheral ground glass radiological pattern. The prognosis of these patients seems favorable, although validation is larger patient cohorts is warranted.

**Supplementary Information:**

The online version contains supplementary material available at 10.1007/s00428-026-04524-z.

## Introduction

Squamous cell carcinoma (SCC) is a malignant epithelial tumour characterized by keratinization, intercellular bridges, or squamous differentiation at immunohistochemistry (IHC) [1].

SCC, the second most common subtype of lung cancer, account for 20% of all lung carcinomas [2] and 30% of non-small cell lung cancers [3].

At CT-scan, lung SCC usually appears as a nodule with sharply demarcated borders or a spiculated mass with distortion of adjacent lung parenchyma [4]. Based on anatomic location of the tumour, SCC can be central or peripheral. Traditionally, SCC has been reported to be mostly centrally located corresponding to two-thirds of lung SCC. However, in recent years an increasing number of peripheral SCC cases (up to 55%) have been reported [3]. Peripheral SCC is defined as a tumour located in the lung periphery from the fourth branching bronchus or, more simply, located in the outer third of the lung at CT-scan [5].

Both central and peripheral type are strongly associated with cigarette smoking [6], nevertheless they do not seem to be biologically related: central SCC are known to arise from bronchial epithelial cells with a multistep progression starting with dysplasia until carcinoma in situ [1], while this sequence has not been proven in peripheral SCC and etiology remain still unknown [7, 8].

Some studies have also found clinicopathological differences between the two SCC subtypes, where peripheral SCC has been associated with older patient age, lower rate of lymph nodes metastasis and lymphatic invasion, lower stage, higher rate of interstitial lung disease and cystic change in adjacent lung parenchyma, more frequent expression of cytokeratin 7 (CK7) IHC. However, no differences in survival were found [5, 7, 9].

In peripheral SCC four main histological pattern have been described: pushing, infiltrative, pseudoalveolar filling and alveolar filling (AF) pattern [10]. This latter is associated with excellent prognosis especially when comprising a major part (> 70%) of the tumor [11], and it has been proposed as the in situ component of peripheral SCC [7].

Moreover, few case-reports in English literature [12–22] describe an unusual growth pattern of peripheral SCC consisting of tumour squamous cells growing on the alveolar walls without their destruction, located under the reactive type II pneumocytes, called lepidic-pagetoid growth pattern of SCC or lepidic SCC. To prevent confusion with the true pagetoid growth of Paget disease, as described in skin and breast pathology and consisting of intraepithelial proliferation of tumoral cells [23, 24], we propose the term “subepithelial” SCC for these type of tumours. Peripheral SCC with this pattern of growth can mimic lung adenocarcinoma both radiologically, showing ground glass appearance at CT-scan, and histologically.

Here we describe a series of 22 peripheral lung SCC with partial subepithelial growth with radiologic and clinical correlation.

## Material and methods

### Patient collection

Lung SCC with subepithelial growth were collected from consultation and regular diagnostic cases at the Amsterdam University Medical Center, Amsterdam, The Netherlands, and Maggiore Hospital of Bologna, Italy, between 1 st January 2018 and 31 st December 2024. Clinical information, overall survival (OS), recurrence free survival (RFS) were retrieved for each patient. The study received ethic committee approval (number 2023.0245 for Amsterdam University Medical Center and number 103/2023/Sper/AOUBo for Maggiore Hospital of Bologna).

### Histology

All cases and previous biopsy when present were reviewed and pathological information with special attention on subepithelial growth were recorded. A representative block with subepithelial growth was selected and 4 µm sections were cut from formalin-fixed paraffin embedded (FFPE) block and stained with Hematoxylin and Eosin (HE), TTF1/p40 double stain and p53 as described below.

**Fig. 1:**
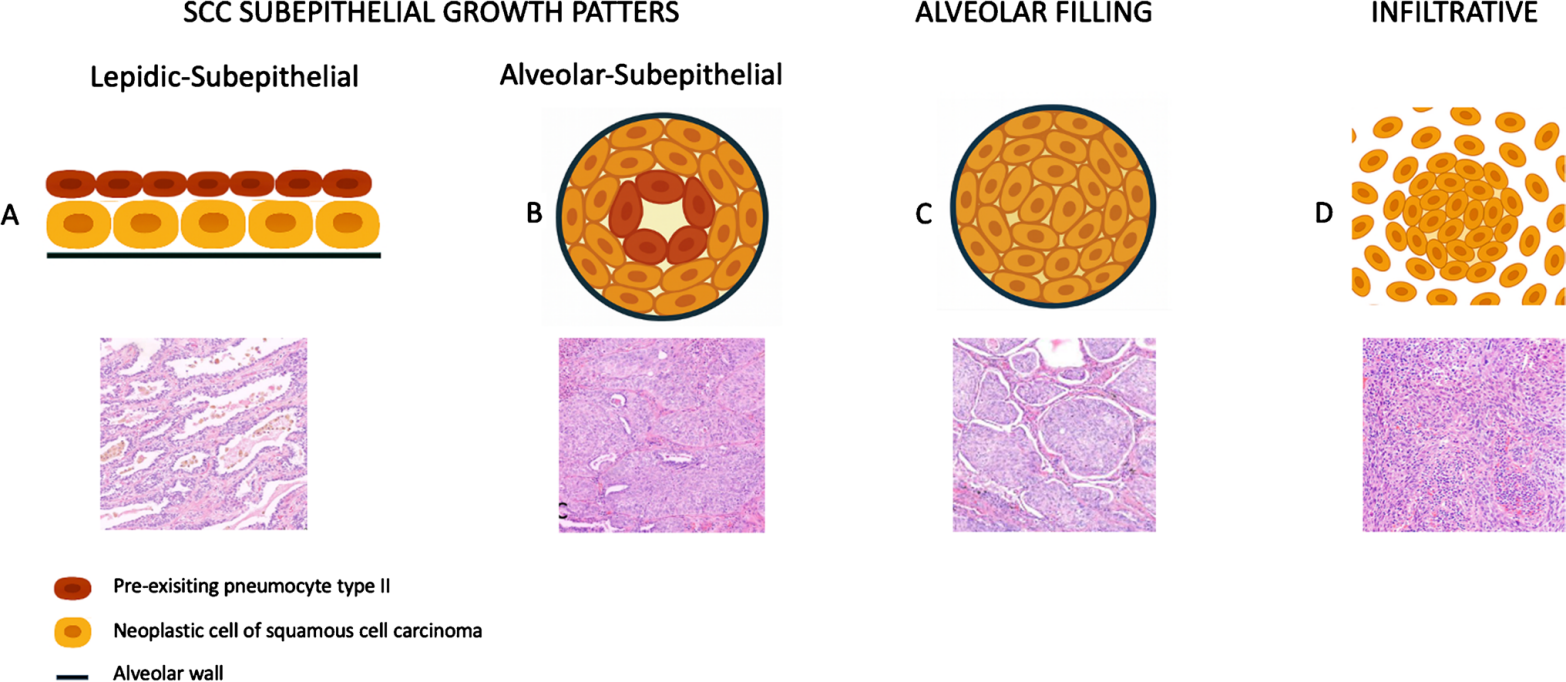
Schematic illustration of subepithelial patterns of squamous cell carcinoma (SCC) (images A and B) together with alveolar-filling SCC (C) and infiltrative SCC (D), with corresponding Haematoxylin–eosin images. Subepithelial growth is characterized by layers of squamous cells beneath normal pneumocytes (**A**) or by nests of tumour cells containing a small, central circular layer of TTF1-positive pneumocytes (**B**). (Graphical illustrations created with Microsoft Copilot)

Subepithelial growth was defined as layers or nests of tumour cells growing along the alveolar septa which are immunoreactive for p40 IHC and covered by normal/reactive pneumocytes type II TTF1 positive. This pattern has been subdivided in lepidic-subepithelial and alveolar-subepithelial [19]: the lepidic-subepithelial pattern is characterized by single tumour cells spreading beneath the pneumocytes while the alveolar-subepithelial is defined by nests of tumour cells within the alveolar space with an inner layer of entrapped pneumocytes. After revision of all slides, the total percentage of following growth patterns of SCC was recorded: lepidic-subepithelial, alveolar-subepithelial, alveolar filling (solid intra-alveolar growth) and infiltrative (Fig. 1).

The CT scan for each case was reviewed and images correlated with histologic sections; the presence of ground glass component of the nodule was recorded. The tumor was defined as peripheral if located in the outer third of the lung at CT-scan [5].

### Immunohistochemistry

IHC for p53 and TTF1-p40 doublestaining were performed on a Ventana Benchmark Ultra immunostainer (Roche, Basel, Switzerland). For detection and visualization of p53, mouse monoclonal anti-P53 diluted 1:2000 in Ventana diluent (double clone DO-7 + BP53-12 catno. MS-738-P, Epredia, Breda, the Netherlands) was used. In brief, antigen retrieval was performed on tissue sections with CC1 (pH85 5424569001 Ventana/Roche) for 48 min at 100 °C and incubation with antibody was for 32 min at 36 °C. Visualization was performed with standard settings for the Optiview DAB detection kit (catno. 06396500001, Roche) and counterstained with Hematoxylin II (catno. 5277965001, Roche). After the immunohistochemical staining, the sections were dehydraded with grades of ethanol and cleared with xylene. All sections were mounted with Tissue Tek® coverslipping film (Sakura Finetek, Alphen aan de Rijn, the Netherlands).

For detection and visualization of the TTF1 and p40 doublestaining, mouse monoclonal anti-TTF1 (clone 8G7G3-1, catno. M3575, Dako/Agilent, Glostrup, Denmark) was used and diluted 1/400 in diluent from Dako/Agilent (catno. S3022, Glostrup, Denmark). Sections were pretreated with CC1 (pH8.5) for 32 min at 100 °C and the antibody was incubated for 64 min at 36 °C. After visualization of TTF1 with standard settings for Optiview Amplification kit (catno. 06396518001, Roche) and Optiview DAB detection kit the slides were pretreated with CC1 for 24 min at 95 °C. Detection and visualization of P40 was performed with a monoclonal antibody P40 (clone BC-28, catno. ACI3066C, Biocare Medical, Concord, USA) diluted 1/100 with Ventana diluent and was incubated for 48 min at 36 °C. Visualization was performed with standard settings of the Amplification kit (catno. 05266114001, Roche) and ultraView Universal Alkaline Phosphatase Red kit Detection Kit (catno. 05269814001, Roche) and sections were counterstained with Hematoxylin II (catno. 5277965001, Roche).

After the immunohistochemical staining, the sections were rapidly dehydraded with grades of ethanol and cleared with xylene. All sections were mounted with Tissue Tek® coverslipping film (Sakura Finetek, Alphen aan de Rijn, the Netherlands).

Six cases underwent Next Generation DNA Sequencing using custom lung panel designed to cover 27 genes [25].

## Results

A total of 22 cases (3 biopsies and 19 resection specimens) with subepithelial growth, corresponding to 21 patients (one patient had in two years two metachronous SCC in separate lobes), were collected. Clinicopathological features are summarized in Table 1.

**Table 1:** Clinicopathological feature of all patients in the study

Case n	Sex	Age	Smoking	ILD	Tumour location	CT appearance	Location	Pleura	Total Diameter (cm)	TNM 8th Edition 2017	p53 IHC	Molecular analysis	Subepithelial pattern SCC		AF SCC (%)	Infiltrative SCC (%)	Previous biopsy	Relapse	FU period (months)
													Lepidic- subepithelial (%)	Alveolar-subepithelial (%)					
1	M	64	yes	yes	LSL	subsolid	peripheral	PL1	1,7	pT2aN0	MU	not performed	0	10	0	90	not performed	no	0
2	F	67	unknown	no	RSL	solid	peripheral	PL0	6	pT3N0	MU	not performed	0	40	0	60	not performed	unknown	unknown
3	M	69	yes	no	LSL	solid	peripheral	PL0	1,2	pT1bN0	MU	not performed	0	70	0	30	not performed	no	38
4	M	63	unknown	no	RIL	solid	peripheral	PL0	2,8	pT1cN0	MU	not performed	0	90	0	10	unknown	unknown	unknown
5	F	74	yes	no	RSL	solid	central	NA	1,5	cT1bN0	MU	TP53 ex7 c.725G > T (p.C242F)	0	100	0	0	unknown	no	4
6	F	70	yes	no	RSL	subsolid	peripheral	PL0	1,4	pT1bN0	MU	TP53 ex5 c.380C > A (p.S127Y)	25	25	0	50	not performed	no	14
7	F	67	unknown	no	LIL	subsolid	peripheral	PL0	1,8	pT1bN0	MU	TP53 ex4 c.313G > T (p.G105C)	70	0	0	30	yes	no	6
8	F	81	unknown	no	LSL	solid	peripheral	PL1	1,1	pT2aN0	MU	not performed	15	5	0	80	not performed	unknown	unknown
9	F	75	yes	no	RIL	subsolid	peripheral	PL0	2,5	pT1cN0	MU	not performed	15	5	0	80	unknown	unknown	unknown
10	F	53	unknown	no	LIL	solid	peripheral	PL0	5,2	pT3N2	MU	not performed	75	15	0	10	unknown	unknown	10
11	M	69	yes	no	LSL	cystic	peripheral	PL0	1,9	pT1bN0	MU	not performed	75	15	0	10	unknown	no	12
12	M	75	yes	no	RSL	solid	peripheral	NA	2	cT1bN0	MU	not performed	100	0	0	0	not performed	no	4
13	M	61	unknown	no	RIL	subsolid	peripheral	PL0	2,1	pT1cN0	MU	not performed	100	0	0	0	unknown	unknown	unknown
14	F	75	former smoker	no	RSL	subsolid	central	NA	2	cT1cN0	WT	KRAS ex4 c.344 G > A (p.G115E) (VUS), STK11 ex2 c.356A > G (p.N119S) (VUS),TP53 ex5 c.475G > C, (p.A159P)	100	0	0	0	unknown	no	24
15	F	63	yes	yes	RSL	solid	peripheral	PL0	2,3	pT1cN0	MU	not performed	60	0	10	30	yes	no	23
16	F	69	former smoker	yes	RIL	solid	peripheral	PL0	1,8	pT1bN0	MU	CDKN2A ex2 c.299C > A, (p.A100D) (VUS), TP53 ex7 c.725G > C (p.C242S)	5	5	20	70	yes	no	17
17	F	71	yes	no	LSL	solid	peripheral	PL0	1,4	pT1bN0	MU	not performed	5	30	30	35	not performed	no	56
18	M	71	yes	yes	RIL	solid	peripheral	PL0	4,5	pT2bN0	MU	not performed	0	10	50	40	not performed	no	38
19	F	63	yes	yes	LIL	subsolid	peripheral	PL0	1,7	pT1bN0	WT	not performed	20	5	55	20	yes	no	33
20	F	51	yes	no	LSL	solid	peripheral	PL0	3,2	pT2aN0	MU	not performed	5	5	80	10	not performed	no	43
21	F	75	former smoker	no	RML	solid	peripheral	PL1	2,8	pT2aN0	MU	no mutations detected	10	10	80	0	not performed	no	34
22	M	75	unknown	no	RIL	solid	peripheral	PL1	2	pT2aN2	MU	not performed	10	10	80	0	unknown	unknown	unknown

M= male, F= female, ILD= interstitial lung disease, RSL= right superior lobe, RIL= right inferior lobe, RML= right medium lobe, LSL= left superior lobe, LIL= left inferior lobe, IHC= immunohistochemistry, MU= mutated pattern, WT= wild-type pattern, VUS= variant of uncertain significance, NA= not applicable, AF= alveolar filling, SCC= squamous cell carcinoma, FU= follow up

The cohort included a higher proportion of female patients (*n* = 13, 62%) with a median age of 69 years (range 51–81 years). Among resected cases, the median tumour diameter was 2 cm (range 1,1–6 cm). In terms of pathological TNM, 11 of resected cases showed pT1 (0 pT1a, 7 pT1b and 4 pT1c), eight cases showed pT2 or pT3 based on TNM classification 8th Edition of 2017. Visceral pleural was infiltrated in four cases (PL1) and nodal metastasis were present in two cases (pN2).

All but two cases (95%) in the study cohort were peripheral SCC.

**Fig. 2:**
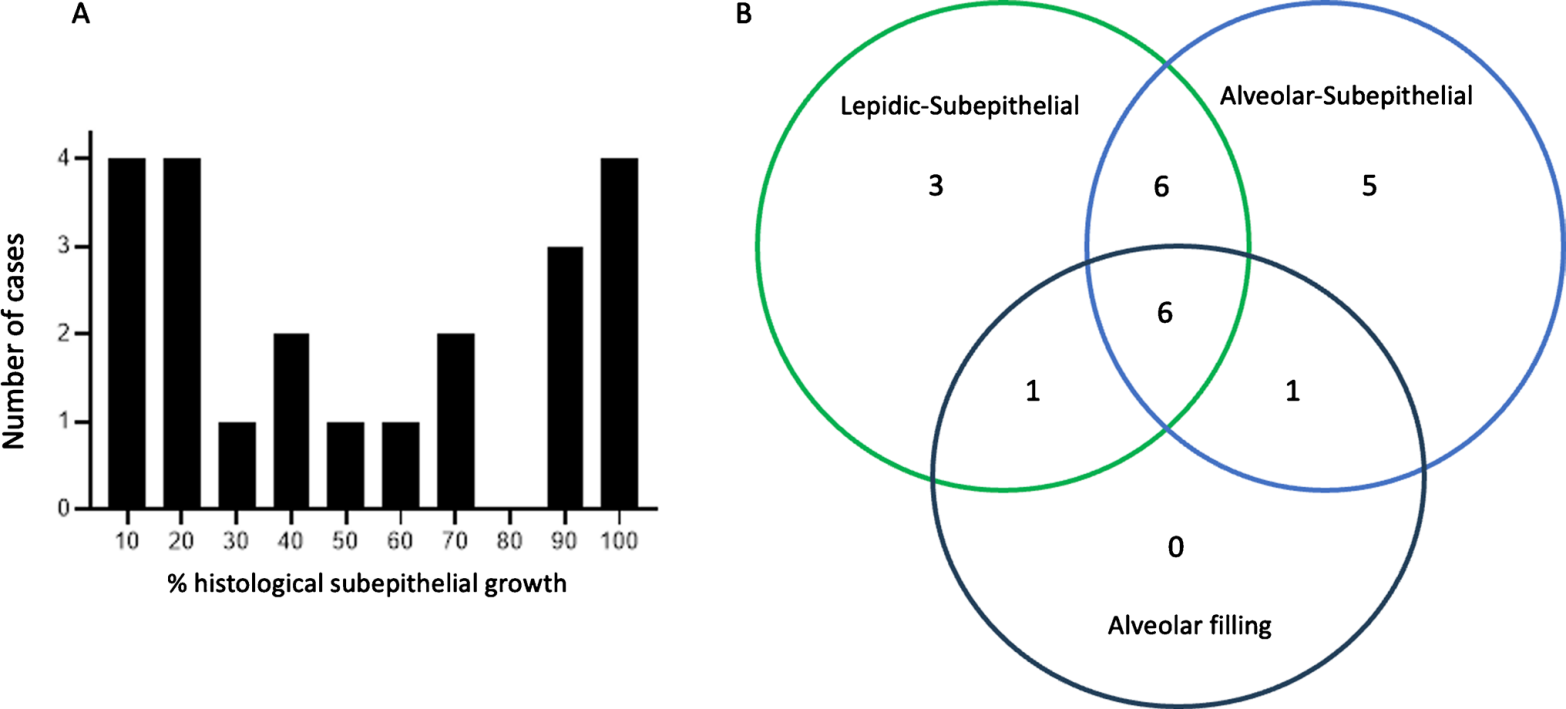
Overlap between the growth patterns of squamous cell carcinoma are represented in the histogram (**A**) and the Venn diagram (**B**)

The distribution of the two subepithelial growth patterns in the cohort was: 100% in four cases (three biopsies and one resection), *n* = 3 cases with 90%, *n* = 11 cases between 20 and 70%, and *n* = 4 with 10% of subepithelial growth (Fig. 2) (Table 1).

**Fig. 3:**
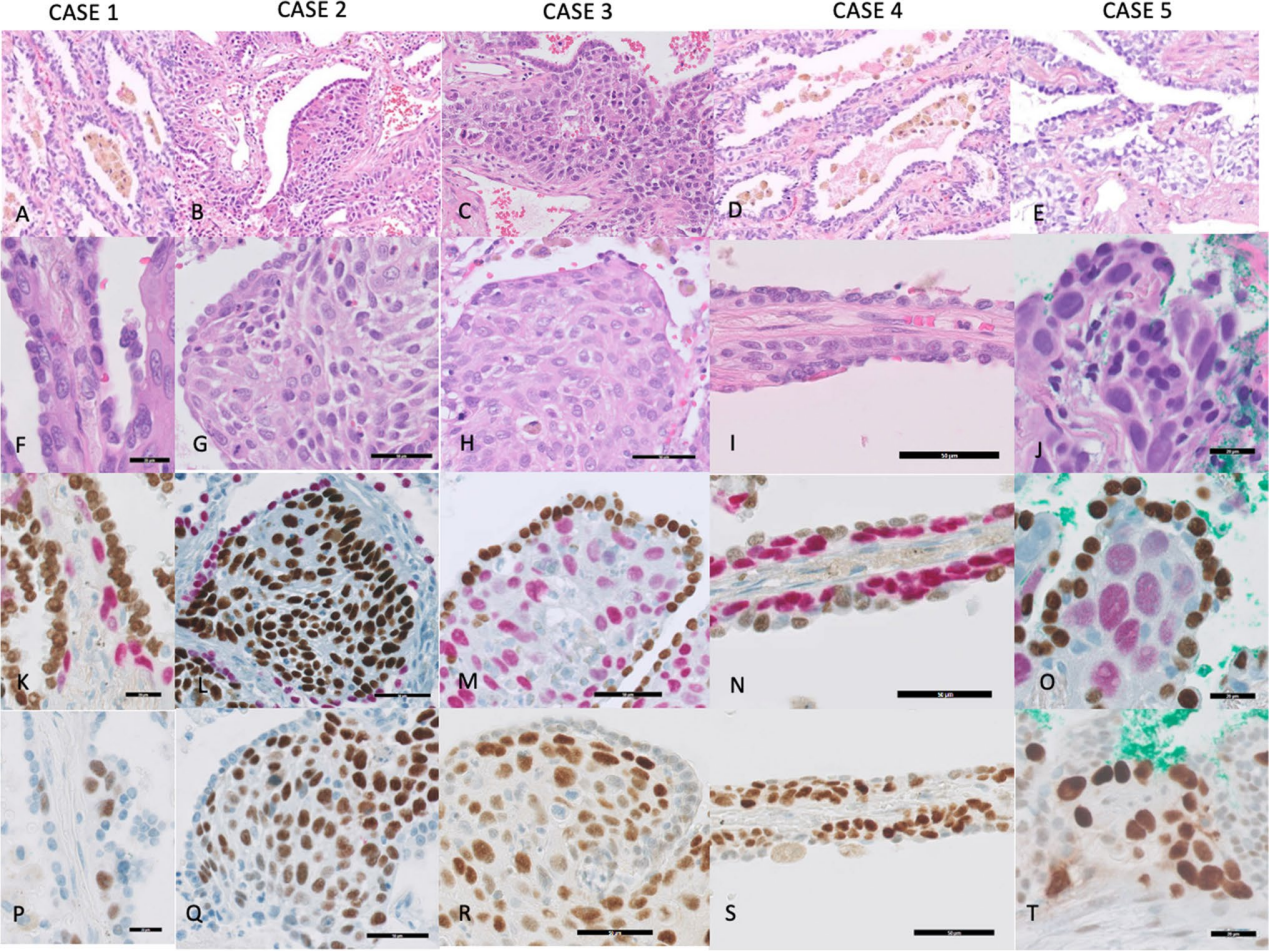
Panel showing five cases of squamous cell carcinoma with subepithelial pattern: cases 1, 4, 5 lepidic-subepithelial pattern, cases 2 and 3 alveolar-subepithelial pattern. (**A**-**J** Haematoxylin–eosin, **K**–**O** double stain TTF1 and p40 with K,M–O TTF1 brown and p40 red, L TTF1 red and p40 brown, P–T p53 immunohistochemistry showing strong immunostaining in squamous carcinoma cells – mutated labeling pattern while non-mutated labeling in TTF1 positive pneumocytes)

In all cases, subepithelial growth was confirmed with double stain TTF1/p40. Most of the cases (20 out of 22, 91%) showed a mutation-type labeling for p53 corresponding to complete absence or strong positivity of immunoreactivity in at least 80% of the tumour cells (Fig. 3). Six cases were sequenced and of those all except one showed a mutation-type pattern of p53 in IHC. In four cases the mutation-type pattern of p53 obtained with IHC was confirmed with molecular sequencing, while one case with wild-type pattern in IHC showed a TP53 gene mutation with sequencing. No mutations were detected in another case that showed a p53 mutation-type pattern in IHC. No other driver mutations were identified.

Lepidic-subepithelial growth was mostly encountered at the periphery of the nodule with a central area of infiltrative SCC. Alveolar-subepithelial growth was located both central and peripheral within the tumor. Lepidic-subepithelial and alveolar-subepithelial growth pattern were both present in 11 cases (50%), while 5 cases (23%) showed only lepidic-subepithelial and 6 cases (27%) only alveolar-subepithelial pattern. Fourteen cases (66%) showed both subepithelial and infiltrative SCC. AF pattern was detected in 8 cases (36%) of which 6 cases were associated with infiltrative SCC (Fig. 2).

Among resection specimens, four cases had preoperative biopsy and one of them showed fully lepidic-subepithelial growth in the biopsy, posing a diagnostic challenge.

More histological pictures of subepithelial SCC are shown in supplementary material (Supplementary Fig. 1–6).

**Fig. 4:**
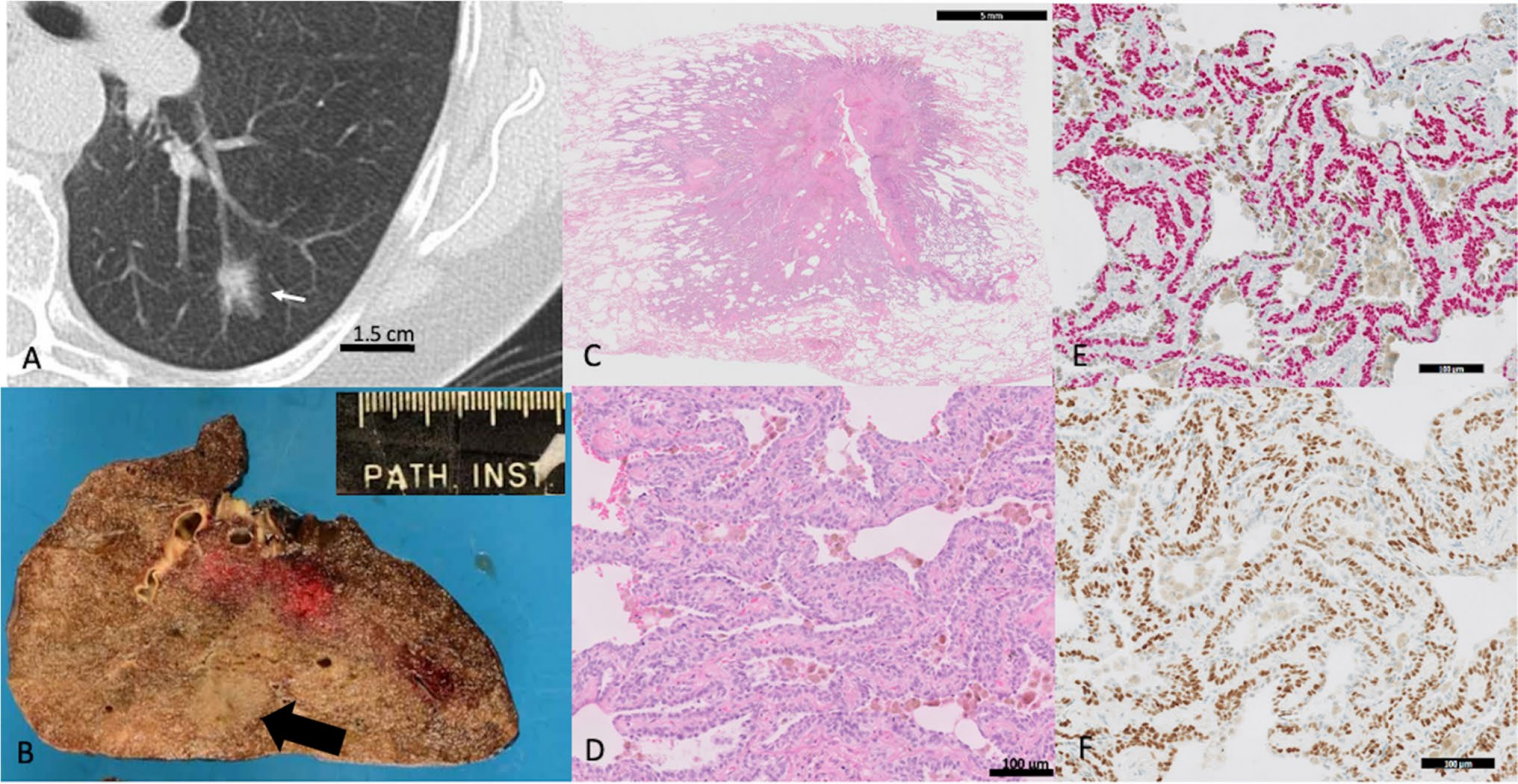
Case of SCC with predominant lepidic-subepithelial pattern. At CT-scan the nodule is subsolid with ground-glass appearance at the periphery (A) which corresponded macroscopically to a grewish nodule (B) and histologically to a nodule with a central area of infiltrative SCC with lepidic-subepithelial pattern of SCC at the edge (C). A high magnification view of the periphery of the nodule shows lepidic-subepithelial pattern of growth (D), confirmed with immunohistochemistry for TTF1/p40 (E). Tumoral cells have a mutated pattern of p53 (F). (C,D Haematoxylin–eosin; E double stain TTF1 brown and p40 red; F p53)

On CT-scan seven cases (33%) showed a subsolid nodule and among them four cases had a subepithelial component ≥50% (Fig. 4); all the other cases except one presented radiologically as a solid nodule; a single case showed a cystic appearance.

Four patients (19%) had concomitant interstitial lung disease.

Follow up information was available in 15 out of 21 patients (71%), none of which experienced recurrence after a median follow-up time of 20 months (range 4–56); three patients died within three years after surgery for non-lung cancer related causes without signs of recurrence.

Given the rarity of this growth pattern, we summarized the clinicopathologic features of cases previously reported in the English literature (Supplementary Table 1).

## Discussion

We systematically describe a SCC series with a rare and under-recognized growth pattern: the subepithelial growth pattern. In our series, as well as in most cases reported in the English literature, subepithelial SCC appear to be associated with good prognosis and in one third of cases the tumour nodules have a ground glass appearance at CT-scan.

Most of SCC are central tumours with one third of cases that are peripherally located. Peripheral SCC were related to a better outcome compared to central SCC at the same stage [1, 7, 8], especially when more than 70% of AF pattern is present [11]. This pattern is associated with excellent prognosis, with no evidence of lymph node metastasis or lymph vascular invasion, and it is mostly peripherally located. Similarly to AF SCC, in our series we observed that SCC with subepithelial growth was mostly peripheral (20 out of 22 cases) with excellent prognosis (none of the patients experienced recurrence nor death of disease); only for 2 cases there were lymph nodal metastasis at diagnosis.

Assuming its favorable prognostic role, AF growth has been proposed to represent a pre-invasive phase in an hypothetical progression to invasive SCC [7, 10] and Guerrieri and colleagues [19] proposed a step-wise progression of SCC from “lepidic-subepithelial” to infiltrative, passing through “alveolar subepithelial” pattern and AF. The origin of the neoplastic squamous cells in the periphery of the lung where type II pneumocytes predominate is very intriguing but cannot be determined with certainty, as no evidence of squamous metaplasia was identified in our series nor in the cases from the literature. Interestingly, based on our data the three patters seem to be biologically related as all cases with AF pattern (*n* = 8) except two showed both lepidic-subepithelial and alveolar-subepithelial growth. Histological evaluation allows assessment of tumour morphology at the time of resection or biopsy; however, it does not permit definitive conclusions regarding tumour oncogenesis or cell of origin. Irrespective of the underlying histogenesis, these tumours appear to exhibit limited destructive potential of the alveolar septa. This observation is consistent with the favorable prognosis reported in our cohort as well as in previous studies [13, 16].

Assuming its favorable prognostic role, treatment decisions could also rely on the presence of the subepithelial component, in both medical and surgical approaches (for example giving the opportunity of a more conservative surgery or denying a chemotherapy) especially in early stages. This is also true for AF SCC: lung SCC with subepithelial and/or AF pattern have a favorable outcome thus both patterns could be considered for treatment decisions. These patterns might be relevant for the potential downstaging of invasive SCC. Larger studies with complete pathological and follow up data are necessary to resolve this matter.

The presence of subepithelial growth was mostly evidenced at the edge of the tumour, giving in some cases (7 out of 22) a ground glass appearance at CT-scan, similarly to adenocarcinoma with lepidic growth, as previously observed [12–14, 16, 18]. The presence of subepithelial growth at the periphery of the tumour should be kept in mind when dealing with biopsies as this could be the only piece of tumour available for diagnosis: IHC for TTF1 and p40 could be very useful in order not to miss subepithelial growth of SCC, especially with biopsies, and to differentiate this tumour with other entities. Major differential diagnosis include lepidic adenocarcinoma, adenosquamous carcinoma and bronchiolar adenoma [26].

Lepidic adenocarcinoma is characterized by neoplastic cells TTF1 positive laying on the alveolar wall, while in lepidic-subepithelial SCC atypical squamous cells (p40 positive and p53 mutated) are encountered under normal or reactive pneumocytes.

In adenosquamous carcinoma both adenocarcinoma and SCC must be encountered (at least 10% of each component) as separate parts of the same tumor or intermixed; on the contrary in subepithelial growth of SCC adenocarcinoma cells are not present. The difference between the reactive pneumocytes type 2 and neoplastic cells can sometimes be challenging and p53 immunohistochemistry can be helpful. Lastly, subepithelial growth of SCC should not be misinterpreted with bronchiolar adenoma, a benign tumour composed of bronchiolar-type cells laying on basal cells [1]. Bronchiolar adenoma is typically peripheral; it shows papillary or glandular architecture, and it is characterized by the presence of cilia on the apex of columnar bronchiolar-type cells in addition to basal cells. Bronchiolar-type cells could be TTF1 positive (especially in distal type B) and basal cells are p40 immunoreactive: in bronchiolar adenoma, as opposed to SCC with subepithelial growth, basal cells are not atypical and show a p53 wild-type pattern in IHC. P53 could, again, be helpful for differentiation of this two entities [26].

Limitations of our study are represented by the limited number of cases in our cohort (even if this is the largest series of subepithelial SCC to our knowledge) and by the small number of cases that underwent comprehensive molecular characterization. However, 91% of cases showed a mutation-type labeling for p53 in IHC suggesting a similar prevalence of TP53 aberrations as described in SCC. More studies with larger cohorts and longer follow up are needed.

In conclusion, subepithelial growth is a rare but important to recognize growth pattern of peripheral SCC with two different models of growth: lepidic-subepithelial and alveolar-subepithelial, with corresponding peripheral ground glass radiological pattern. Subepithelial growth should not be mistaken for adenosquamous carcinoma or lepidic adenocarcinoma. Subepithelial growth of SCC seems to be associated with low pT stage and favorable clinical outcomes, although larger studies are needed to confirm these associations.

Legends of Supplementary Figures

Suppl. Figure 1 Examples of lepidic-subepithelial growth pattern (case A-B) and alveolar- subepithelial growth of squamous cell carcinoma (C-D). (A, C Haematoxylin–eosin; B, D double stain TTF1 brown and p40 red).

Suppl. Figure 2 Two cases of squamous cell carcinoma with lepidic-subepithelial growth at the edge of the tumour. At low power (A and E) the lepidic-subepithelial growth mimic the lepidic growth of adenocarcinoma. A-D Case 1, E–H Case 2. C-D and G-H correspond to a high magnification view of the lepidic-subepithelial component of SCC. (A, E, G Haematoxylin–eosin; B, C, D, F, H double stain TTF1 brown and p40 red).

Suppl. Figure 3 Squamous cell carcinoma with predominant lepidic-subepithelial growth. Low power view (A and B) and high-power view (C and D). Image C shows the tumour growing mostly as scattered cells under pneumocytes (lepidic-subepithelial pattern) with some focal areas of alveolar-subepithelial growth. The tumoral cells are strongly immunoreactive for p53. (A Haematoxylin–eosin; B, C double stain TTF1 brown and p40 red; D p53).

Suppl. Figure 4 Alveolar-subepithelial squamous cell carcinoma (SCC). Low power view (image A) and high magnification (B, C, D) of the tumour. Tumour nests of SCC grow within the alveolar spaces under normal pneumocytes TTF1 positive. (A,B,C Haematoxylin–eosin; D double stain TTF1 brown and p40 red).

Suppl. Figure 5 Case of SCC with both lepidic-subepithelial and alveolar-subepithelial growth pattern at the edge of the tumour. C and D correspond to high magnification areas at the periphery of the tumour. (A Haematoxylin–eosin; B,C,D double stain TTF1 brown and p40 red).

Suppl. Figure 6 Biopsies of lung tumours showing squamous cell carcinoma (SCC) growing under normal pneumocytes. Images A and B correspond to a case of lepidic-subepithelial pattern of SCC, highlighted by p40 positive cells; pictures C and D show the biopsy of a case with alveolar-subepithelial SCC. (A,C Haematoxylin–eosin; B,D double stain TTF1 brown and p40 red).

## Supplementary Information

Below is the link to the electronic supplementary material.

ESM 1 (21.5 KB)

ESM 2 (PNG 11.6 MB)

High resolution image (TIF 6.69 MB)

ESM 3 (PNG 1.65 MB)

ESM 4 (PNG 1.98 MB)

ESM 5 (PNG 2.93 MB)

ESM 6 (PNG 1.99 MB)

ESM 7 (PNG 2.00 MB)

## References

[1] *Thoracic Tumours*, 5th Edition. Lyon: International Agency for Research on Cancer: WHO Classifications of Tumours, 2021.

[2] Barta JA, Powell CA, Wisnivesky JP (2019) Global epidemiology of lung cancer. Ann Glob Health 85(1). 10.5334/aogh.2419 30741509 10.5334/aogh.2419 PMC6724220

[3] Krimsky W et al (2016) The changing anatomic position of squamous cell carcinoma of the lung - a new conundrum. J Community Hosp Intern Med Perspect 6(6). 10.3402/jchimp.v6.33299 27987285 10.3402/jchimp.v6.33299 PMC5161782

[4] Takahashi M, Nitta N, Takazakura R, Nishimoto Y, Furukawa A, Murata K (2003) Many faces of squamous cell carcinoma of the lung: its wide spectrum of radiological findings. Curr Probl Diagn Radiol 32(2):45–65. 10.1067/mdr.2003.120008 12658263 10.1067/mdr.2003.120008

[5] Kosaka T et al (2020) Clinicopathological features of small-sized peripheral squamous cell lung cancer. Mol Clin Oncol 12(1):69–74. 10.3892/mco.2019.1951 31814978 10.3892/mco.2019.1951 PMC6888248

[6] Sakurai H, Asamura H, Watanabe S, Suzuki K, Tsuchiya R (2004) Clinicopathologic features of peripheral squamous cell carcinoma of the lung. Ann Thorac Surg 78(1):222–227. 10.1016/j.athoracsur.2004.01.029 15223433 10.1016/j.athoracsur.2004.01.029

[7] Funai K et al (2003) Clinicopathologic characteristics of peripheral squamous cell carcinoma of the lung. Am J Surg Pathol 27(7):978–984. 10.1097/00000478-200307000-00013 12826890 10.1097/00000478-200307000-00013

[8] Sung YE, Cho U, Lee KY (2020) Peripheral type squamous cell carcinoma of the lung: clinicopathologic characteristics in comparison to the central type. J Pathol Transl Med 54(4):290–299. 10.4132/jptm.2020.05.04 32544984 10.4132/jptm.2020.05.04 PMC7385267

[9] Saijo T et al (2006) Differences in clinicopathological and biological features between central-type and peripheral-type squamous cell carcinoma of the lung. Lung Cancer 52(1):37–45. 10.1016/j.lungcan.2005.12.006 16497410 10.1016/j.lungcan.2005.12.006

[10] Yousem SA (2009) Peripheral squamous cell carcinoma of lung: patterns of growth with particular focus on airspace filling. Hum Pathol 40(6):861–867. 10.1016/j.humpath.2008.11.008 19269005 10.1016/j.humpath.2008.11.008

[11] Watanabe Y et al (2011) Alveolar space filling ratio as a favorable prognostic factor in small peripheral squamous cell carcinoma of the lung. Lung Cancer 73(2):217–221. 10.1016/j.lungcan.2010.12.001 21216489 10.1016/j.lungcan.2010.12.001

[12] Terada Y et al (2017) Squamous cell carcinoma of the lung showing a ground glass nodule on high-resolution computed tomography associated with pneumoconiosis: a case report. Surg Case Rep 3(1). 10.1186/s40792-017-0384-1 28963659 10.1186/s40792-017-0384-1 PMC5622012

[13] Sakaizawa T et al (2015) A case of pulmonary squamous cell carcinoma revealed ground glass opacity on computed tomography. J Thorac Oncol 10(8):1229–1230. 10.1097/JTO.0000000000000462 26200279 10.1097/JTO.0000000000000462

[14] Kobayashi H, Nagao H, Kanoh S, Motoyoshi K, Ozeki Y, Aida S (2006) Squamous cell carcinoma of the lung spreading along the alveolar walls with a bubblelike appearance on HRCT. J Thorac Imaging 21(1):57–59. 10.1097/01.rti.0000204400.63644.88 16538160 10.1097/01.rti.0000204400.63644.88

[15] Nakanishi K, Kawai T, Suzuki M, Torikata C (1996) Bronchogenic squamous cell carcinomas with invasion along alveolar walls. Histopathology 29(4):363–368. 10.1111/j.1365-2559.1996.tb01420.x 8910044 10.1111/j.1365-2559.1996.tb01420.x

[16] Iguchi H, Murata S-I, Kawago M, Hirai Y, Kojima F, Nishimura Y (2021) Unique clinicopathological characteristics of pulmonary squamous cell carcinoma with part-solid nodule. Respirol Case Rep 9(1). 10.1002/rcr2.692 33251014 10.1002/rcr2.692 PMC7678643

[17] Pääkkö P, Risteli J, Risteli L, Autio-Harmainen H (1990) Immunohistochemical evidence that lung carcinomas grow on alveolar basement membranes. Am J Surg Pathol 14(5):464–473. 10.1097/00000478-199005000-00006 2158243 10.1097/00000478-199005000-00006

[18] Atsumi J et al (2013) A peculiar squamous dysplastic lesion presenting as a ground-glass opacity: a case report. Eur Respir J 41(5):1228–1230. 10.1183/09031936.00098112 23633612 10.1183/09031936.00098112

[19] Guerrieri C, Lindner M, Sesti J, Chakraborti A, Hudacko R (2022) Pulmonary squamous cell carcinoma with a lepidic-pagetoid growth pattern. Pathologica 114(4):304–311. 10.32074/1591-951X-450 36136898 10.32074/1591-951X-450 PMC9624129

[20] A. Del Gobbo et al. (2016) ‘Pulmonary squamous cell carcinoma with lepidic growth pattern: new insights into lung cancer classification’, vol. 9, no. 7, pp. 7668–7673.

[21] Nakao A, Hamasaki M, Waseda R, Watanabe K (2018) Squamous cell carcinoma appearing as a multi-cystic lesion. Intern Med 57(19):2907–2909. 10.2169/internalmedicine.0050-17 29709924 10.2169/internalmedicine.0050-17 PMC6207822

[22] Durra H, Flieder DB (2012) Peripheral squamous cell carcinoma of the lung: potential pitfalls in biopsy interpretation. Pathol Case Rev 17(5):211. 10.1097/PCR.0b013e318270ab43

[23] *Breast Tumours*, 5th Edition. (2019) WHO Classifications of Tumours Editorial Board.

[24] *Skin Tumours*, 5th Edition. (2025) WHO Classifications of Tumours Editorial Board.

[25] Janssen J et al (2025) Performance and considerations in the use of diagnostic mutation panels for clonality testing in non-small-cell lung carcinoma. ESMO Open 10(5). 10.1016/j.esmoop.2025.105072 40373350 10.1016/j.esmoop.2025.105072 PMC12141053

[26] Chang JC et al (2018) Bronchiolar adenoma: expansion of the concept of ciliated muconodular papillary tumors with proposal for revised terminology based on morphologic, immunophenotypic, and genomic analysis of 25 cases. Am J Surg Pathol 42(8):1010–1026. 10.1097/PAS.0000000000001086 29846186 10.1097/PAS.0000000000001086 PMC8063713

