# Supplementary material for: Lepidic and alveolar subepithelial squamous cell carcinoma: expansion of the concept of peripheral squamous cell carcinoma with proposal for revised terminology based on morphologic, immunophenotypic, and clinical analysis of 22 cases
Source: Virchows Arch. 2026 Apr 23;489(1):27–35. doi: 10.1007/s00428-026-04524-z (PMC13368955; doi:10.1007/s00428-026-04524-z)
Supplement: Supplementary file 1 — (21.5 KB) [file 428_2026_4524_MOESM1_ESM.docx]

| **Case n.** | **Sex** | **Age** | **Smoking (pack /year)** | **ILD** | **Tumour location** | **CT appearance** | **Lung location** | **Pleura** | **Total Diameter (cm)** | **TNM 8th Edition 2017** | **p53** | **Pagetoid pattern of SCC** | **Pagetoid pattern of SCC (%)** | **Alveolar Filling SCC** | **Infiltrative SCC** | **Relapse** | **Biopsy before surgery** | **FU period (months)** | **Reference** |
| --- | --- | --- | --- | --- | --- | --- | --- | --- | --- | --- | --- | --- | --- | --- | --- | --- | --- | --- | --- |
| 1 | M | 62 | Yes (pack/year NR) | No | RML | Solid | Peripheral | NR | 4.9 | NR | NR | Present (lepidic) | NR | NR | Present | NR | Present, (suggestive for ADC) | NR | Durra & Flieder 2012 |
| 2 | M | 68 | Former smoker | No | LSL | Solid | Peripheral | NR | 1.2 | pT1a N0 | NR | Present (lepidic and pseudoglandular) | NR | Present, focal | Present | NR | Absent | 1 | Guerrieri et al. 2022 |
| 3 | F | 61 | Never smoker | No | LIL | GGO | Peripheral | NR | 1.5 | pTis N0 (squamous dysplasia) | NR | Present | 100 | NR | Absent | NR | Absent | NR | Atsumi et al. 2013 |
| 4 | M | 54 | Yes (70 pack/year) | No | RSL | Subsolid | Peripheral | NR | 2.5 | NR | NR | Present | 60 | NR | Present | None | Present | 36 | Kobayashi et al. 2006 |
| 5 | F | 68 | Never smoker | No | RSL | Subsolid | Peripheral | NR | 4.3 | NR | NR | Present (lepidic pattern) | NR | NR | Present | None | Absent | 48 | Sakaizawa et al. 2015 |
| 6 | M | 77 | Yes (35 pack/year) | Pneumoconiosis | RIL | Subsolid | Peripheral | PL0 | 2.4 | pT1a N0 M0 | NR | Present | NR | NR | Present | None | Absent | 19 | Terada et al. 2017 |
| 7 | M | 73 | Yes (pack/year NR) | No | LSL | Solid | Peripheral | NR | 1.3 | NR | NR | Present (lepidic) | NR | Absent | Present | NR | Absent | NR | Del Gobbo et al. 2016 |
| 8 | M | 70 | Yes (50 pack/year) | No | RIL | Subsolid | Peripheral | NR | 2.5 | NR | NR | Present (lepidic) | NR | NR | Present | NR | Absent | 12 | Iguchi et al. 2021 |
| 9 | M | 68 | Yes (20 pack/year) | No | RSL | Solid | Peripheral | NR | 10.5 | NR | NR | Present (lepidic and pseudoglandular) | 45 | NR | Present | Yes | Present (SCC) | 7 | Nakanishi et al. 1996 - Case 1 |
| 10 | M | 69 | Yes (49 pack/year) | No | RML | Subsolid | Peripheral | NR | 3.0 | NR | NR | Present (lepidic and pseudoglandular) | 35 | NR | Present | None | Absent | 40 | Nakanishi et al. 1996 - Case 2 |
| 11 | M | 76 | NR | No | LIL | Cystic | NR | NR | NR | NR | NR | Present | NR | NR | Present | NR | NR | NR | Nakao et al, 2018 |

Legend Supplementary Table 1

Clinicopathological feature of patients with pagetoid squamous cell carcinoma reported in English literature (M= male, F= female, NR= not reported, ILD= interstitial lung disease, RSL= right superior lobe, RIL= right inferior lobe, RML= right medium lobe, LSL= left superior lobe, LIL= left inferior lobe, GGO= ground glass opacity, SCC = squamous cell carcinoma, ADC= adenocarcinoma, FU= follow up).
