# Supplementary figures and images for: Lepidic and alveolar subepithelial squamous cell carcinoma: expansion of the concept of peripheral squamous cell carcinoma with proposal for revised terminology based on morphologic, immunophenotypic, and clinical analysis of 22 cases
Source: Virchows Arch. 2026 Apr 23;489(1):27–35. doi: 10.1007/s00428-026-04524-z (PMC13368955; doi:10.1007/s00428-026-04524-z)

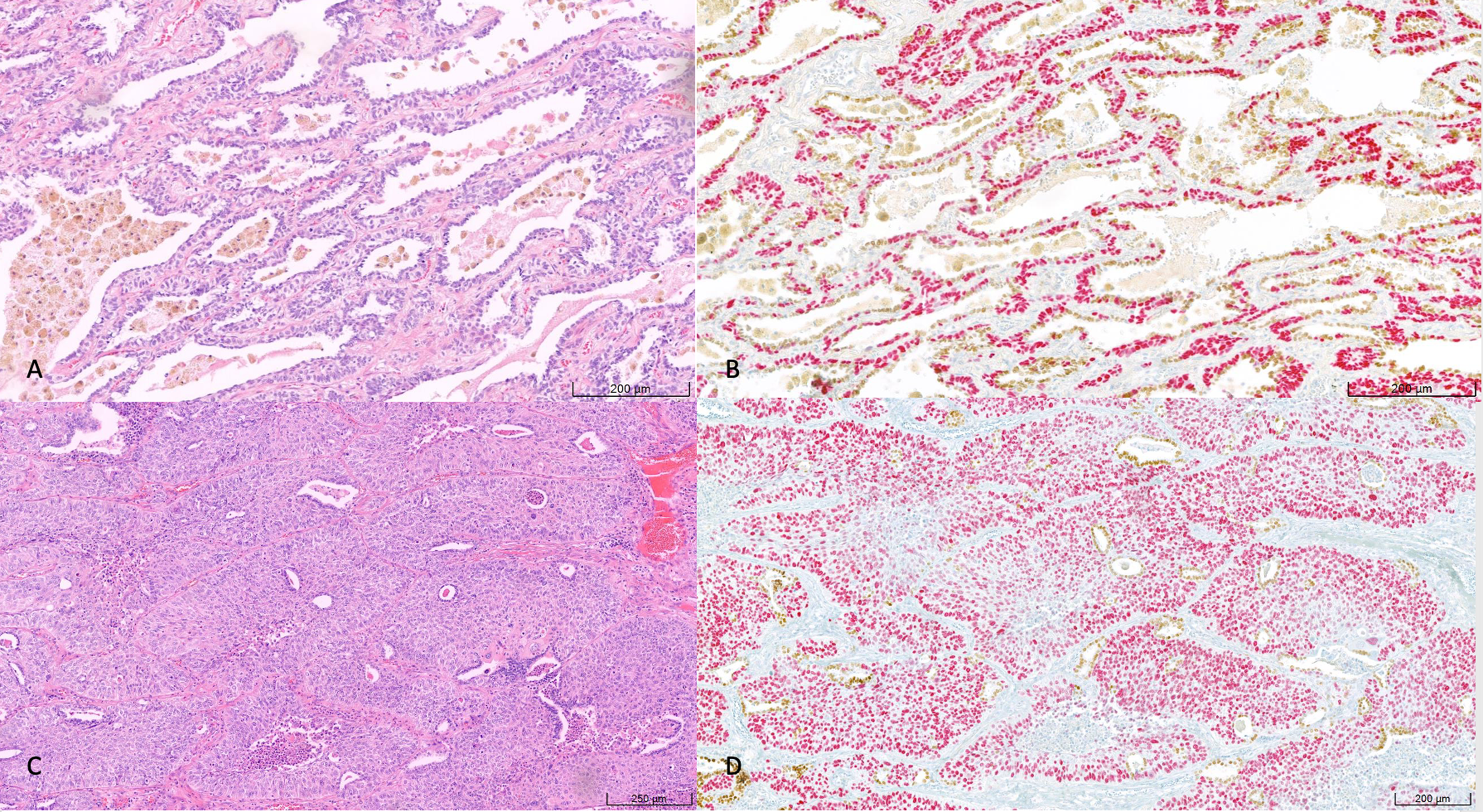

Supplement: Supplementary file 2 — (PNG 11.6 MB) [file 428_2026_4524_Fig5_ESM.png]

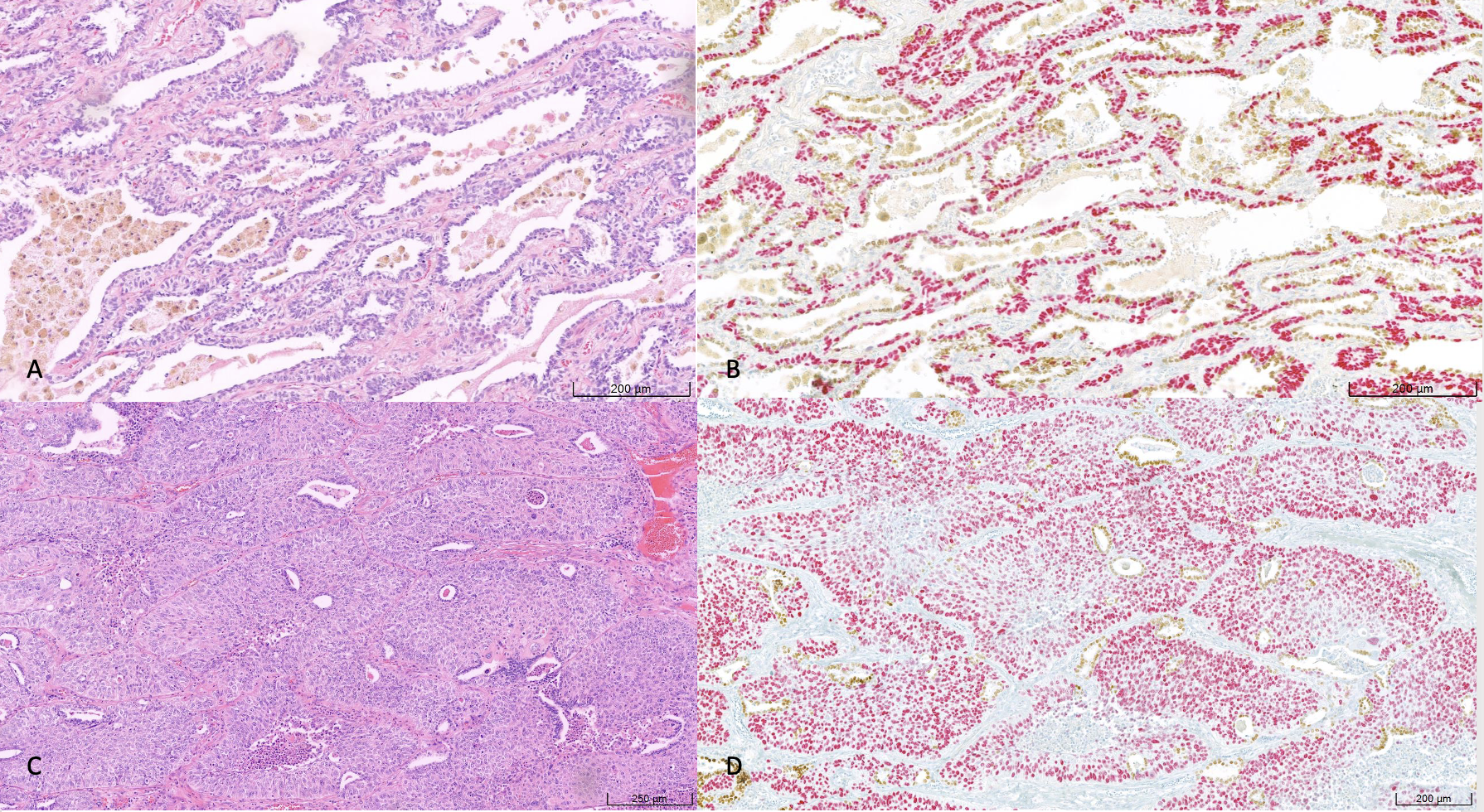

Supplement: Supplementary file 3 — High resolution image (TIF 6.69 MB) [file 428_2026_4524_MOESM2_ESM.tiff]

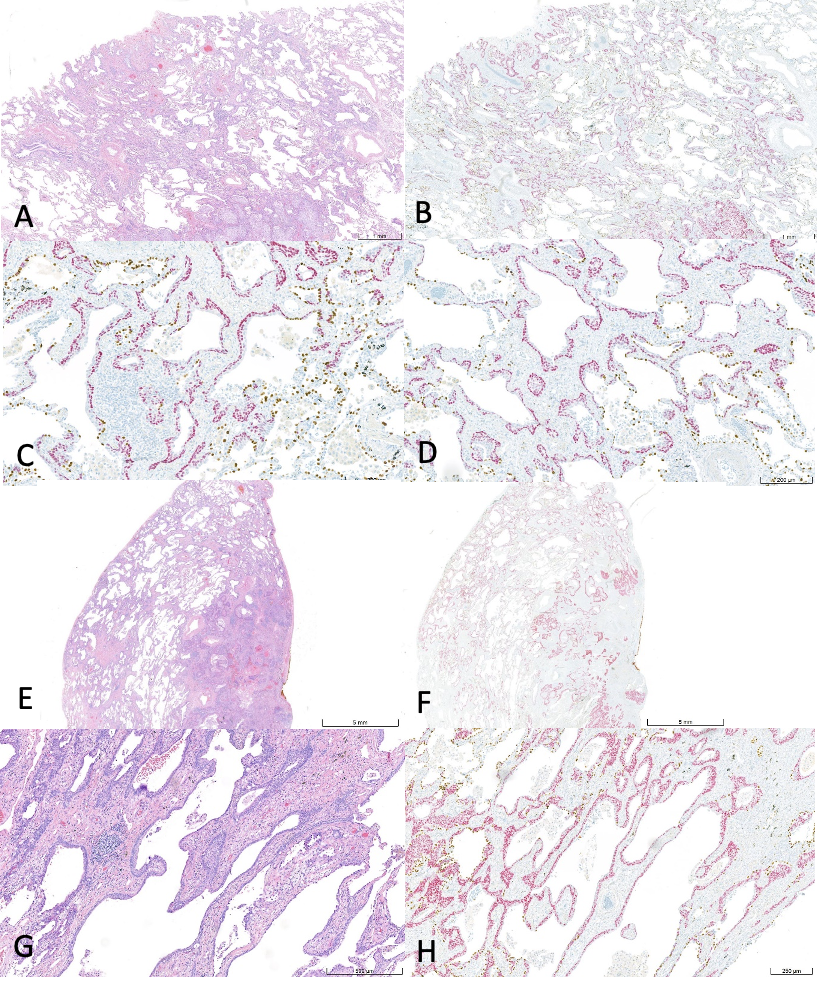

Supplement: Supplementary file 4 — (PNG 1.65 MB) [file 428_2026_4524_MOESM3_ESM.png]

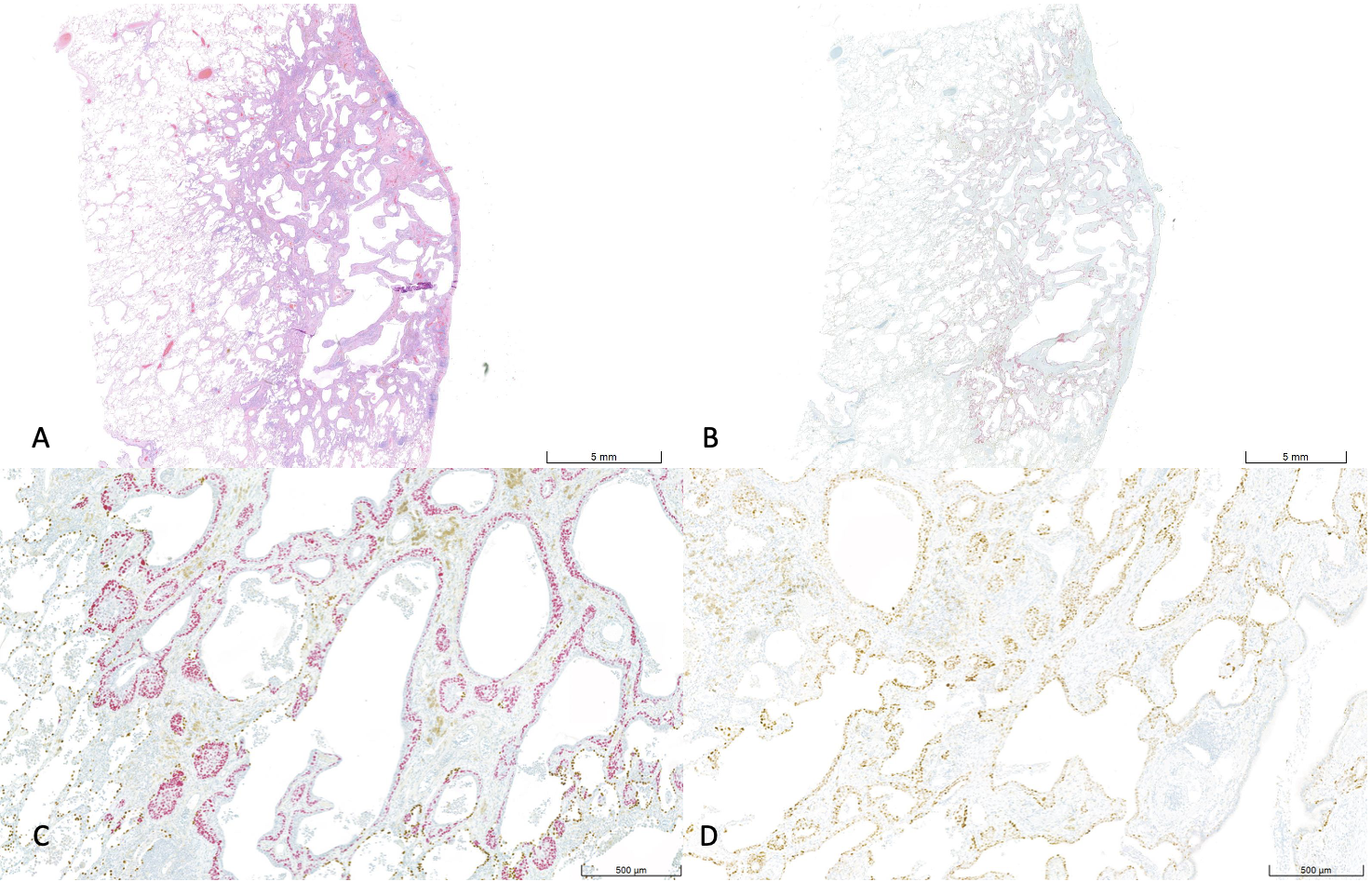

Supplement: Supplementary file 5 — (PNG 1.98 MB) [file 428_2026_4524_MOESM4_ESM.png]

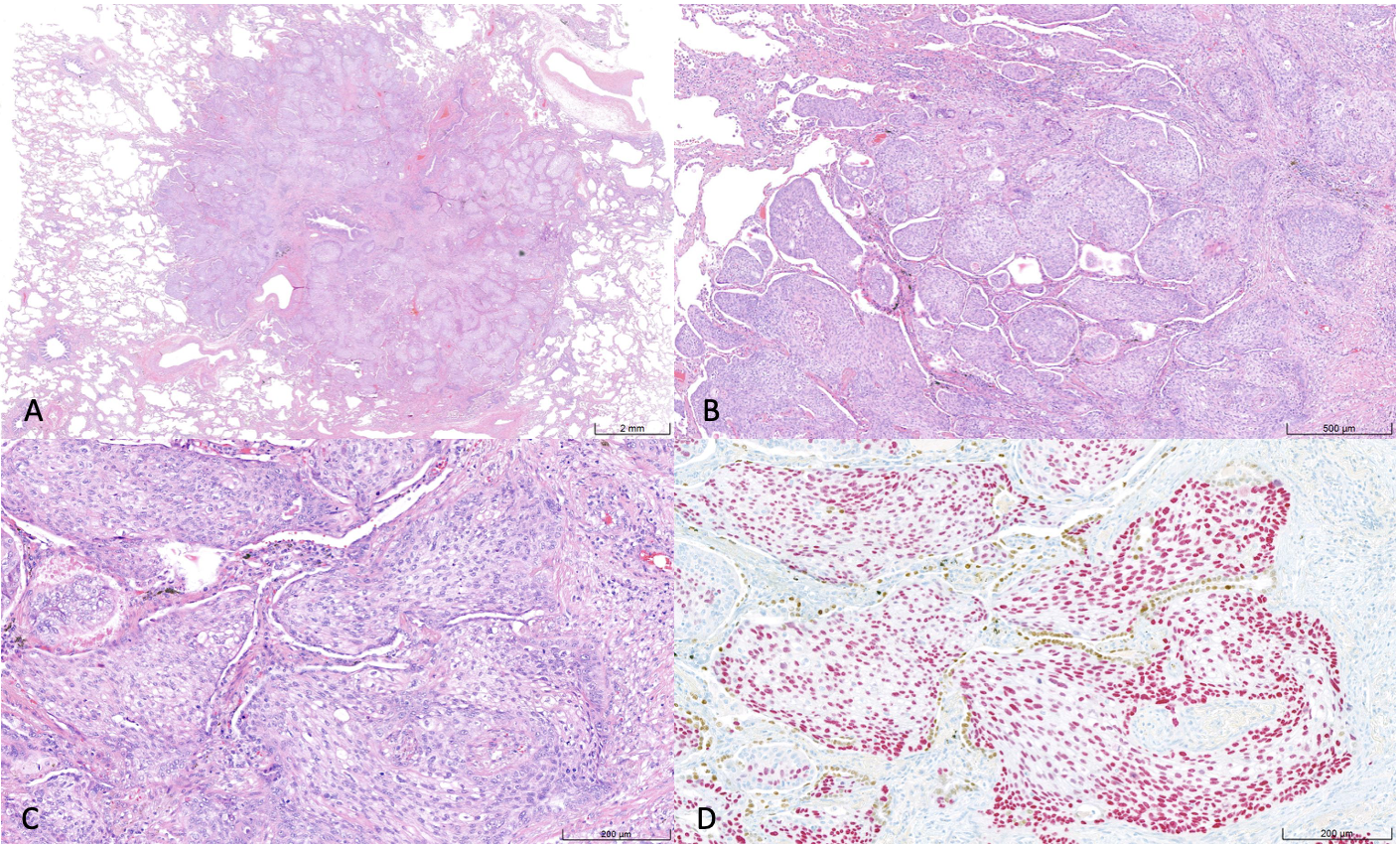

Supplement: Supplementary file 6 — (PNG 2.93 MB) [file 428_2026_4524_MOESM5_ESM.png]

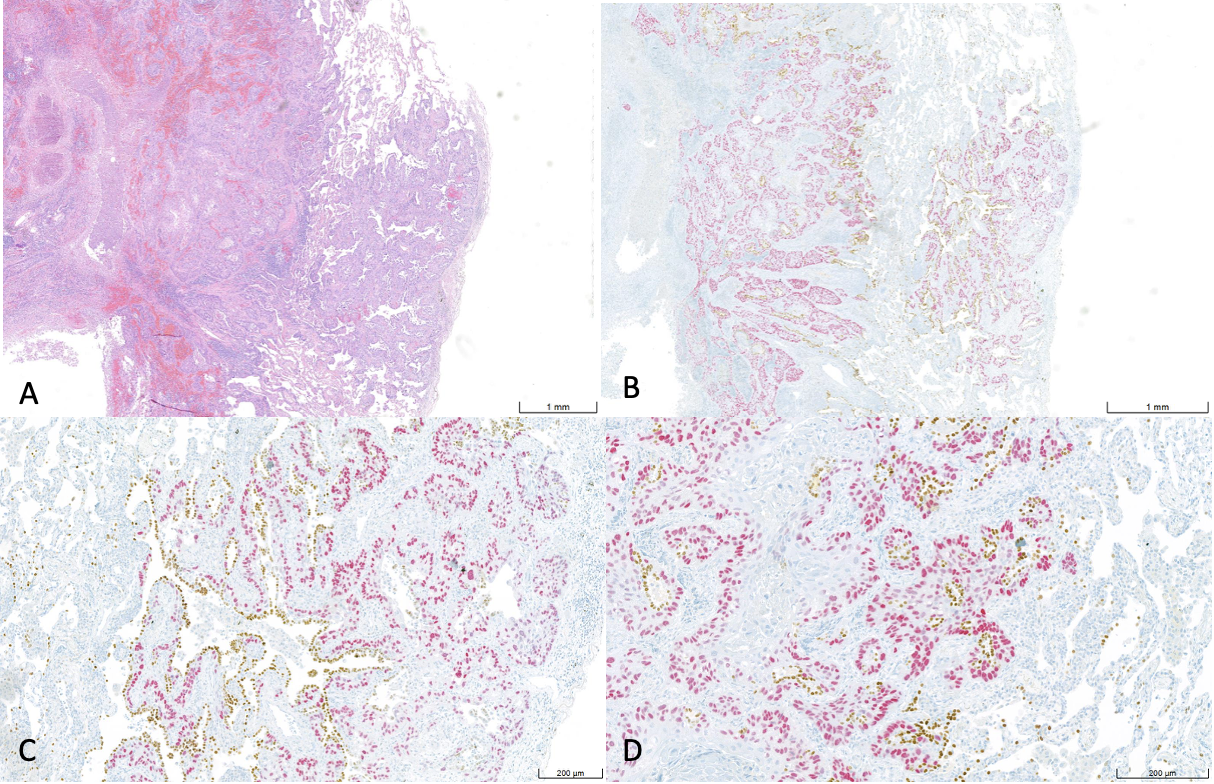

Supplement: Supplementary file 7 — (PNG 1.99 MB) [file 428_2026_4524_MOESM6_ESM.png]

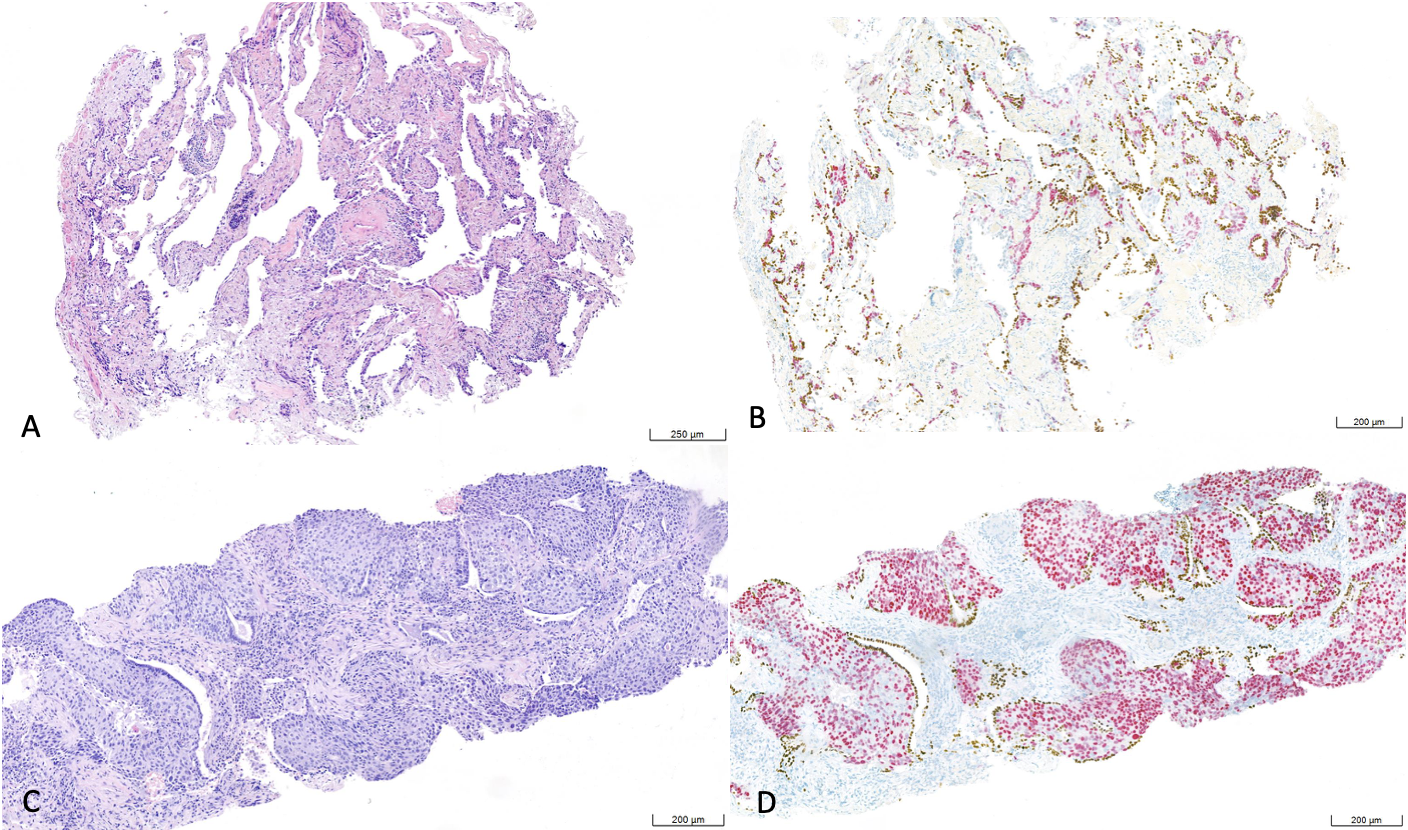

Supplement: Supplementary file 8 — (PNG 2.00 MB) [file 428_2026_4524_MOESM7_ESM.png]
